# The SEM-4 Transcription Factor Is Required for Regulation of the Oxidative Stress Response in *Caenorhabditis elegans*

**DOI:** 10.1534/g3.120.401316

**Published:** 2020-07-24

**Authors:** Adilya Rafikova, Queenie Hu, Terrance J. Kubiseski

**Affiliations:** * Department of Biology, York University, Toronto, Ontario, Canada M3J 1P3; † Program in Neuroscience, York University, Toronto, Ontario, Canada M3J 1P3

**Keywords:** *Caenorhabditis elegans (C. elegans)*, oxidative stress, genetics, gene expression, transcription factor, SEM-4, Splat-like (SALL) family of transcription factors

## Abstract

Oxidative stress causes damage to cells by creating reactive oxygen species (ROS) and the overproduction of ROS have been linked to the onset of premature aging. We previously found that a *brap-2* (BRCA1 associated protein 2) mutant significantly increases the expression of phase II detoxification enzymes in *C. elegans*. An RNAi suppression screen to identify transcription factors involved in the production of *gst-4* mRNA in *brap-2* worms identified SEM-4 as a potential candidate. Here, we show that knockdown of *sem-4* suppresses the activation of *gst-4* caused by the mutation in *brap-2*. We also demonstrate that *sem-4** is* required for survival upon exposure to oxidative stress and that SEM-4 is required for expression of the transcription factor SKN-1C. These findings identify a novel role for SEM-4 in ROS detoxification by regulating expression of SKN-1C and the phase II detoxification genes.

The nematode *C. elegans* has well-defined stress defense systems for protection from toxic compounds (Van Raamsdonk and Hekimi 2010) and it offers a suitable model to dissect the gene regulatory network involved in the expression of stress response genes. Attention has been given to the transcription factors DAF-16/FOXO and SKN-1/Nrf2 due to their roles in regulating transcription in response to oxidative stress and lifespan extension in *C. elegans* (Kenyon *et al.* 1993; Murphy *et al.* 2003; An and Blackwell 2003; Blackwell *et al.* 2015). These factors regulate the transcription of detoxification genes such as *sod-3* and *gst-4*, to regulate levels of ROS (Oliveira *et al.* 2009; Wang *et al.* 2010; Shore and Ruvkun 2013). Although our understanding of the oxidative stress response has improved, identification of transcription factors involved in this process and the control of their activities remains to be fully characterized.

Mammalian Brap2 (Brca1 a ssociated binding p rotein 2; Brap as listed in the HUGO database) is a Ras-responsive E3 ubiquitin ligase that functions as a modulator of the Ras signaling pathway by facilitating activation of Erk upon cell stimulation (Ory and Morrison 2004; Matheny and White 2006, 2009) and also appears to act as a cytoplasmic retention protein for a number of proteins (Li *et al.* 1998; Asada *et al.* 2004; Chen *et al.* 2009; Davies *et al.* 2013). Previously we have shown that the *C. elegans* homolog *brap-2* loss of function mutant increases the MAP kinase activity of PMK-1 that leads to the increased expression of a wide range of phase II detoxification genes such as the glutathione S-transferases *gst-4*, *gst-7*, *gst-10*, and *gsto-2*, the dehydrogenase *dhs-8*, the gamma glutamylcysteine synthetase *gcs-1* and the UDP-glucurononsyl transferase *ugt-13* (Hu *et al.* 2017). We also performed an RNAi screen and showed that BRAP-2 regulates the transcription factor SKN-1 for the induction of phase II detoxification genes. SKN-1 is the *C. elegans* Nrf2 ortholog and, along with DAF-16, is a major transcriptional regulator of stress response in nematodes (Blackwell *et al.* 2015). Here we investigate the possibility that SEM-4, another candidate from the RNAi screen, is required for the oxidative stress response in the *brap-2* mutant and during oxidative conditions.

SEM-4 (Sex Muscle abnormal 4) is a zinc finger (ZnF) containing transcription factor in *C. elegans* that is involved in neuronal, vulval and body wall muscle cell fate (Basson and Horvitz 1996). The *sem-4* mutant worms are egg laying defective due to the transformation of sex myoblasts to body muscle cells. In addition to the development of a proper vulva, expression of SEM-4 has been shown to be important for the proper development and function of motor neurons and touch receptor neurons in the animal (Basson and Horvitz 1996; Toker *et al.* 2003; Kagias *et al.* 2012). SEM-4 is part of the NODE complex (CEH-6, EGL-27, SOX-2 (SRY (sex determining region Y)-box 2) and SEM-4) that activates EGL-5, and allows transformation of the Y cell (rectal epithelial cell) into PDA cell (motor neuron) (Kagias *et al.* 2012).

Here, we report that SEM-4 has novel role in the SKN-1 dependent oxidative stress response as it promotes expression of phase II detoxification genes, likely through regulating *skn-1c* gene expression, and we demonstrate that *sem-4** is* required for survival upon exposure to oxidative stress. This work provides evidence that SEM-4 has a novel role in the complex regulatory network that controls expression of genes involved in the oxidative stress response.

## Methods and Materials

### C. elegans strains

All *C. elegans* strains were maintained as described by Brenner (Brenner 1974). Double mutant strains were generated according to standard protocols. Unless stated otherwise, worm strains were provided by the *Caenorhabditis* Genetics Center (CGC, University of Minnesota). Strains used in this study are listed in Table S1.

### Fluorescence microscopy

L4 *gst-4p*::*gfp* expressing worms were anesthetized using 2 mM Levamisole (Sigma L9756) and mounted on 2% agarose pad. Images of fluorescent worms were taken using a Zeiss LSM 700 confocal laser-scanning microscope with Zen 2010 Software.

### RNA isolation and quantitative real time PCR

Worm RNA isolation, quantitative RT-PCR and data analysis were executed as described previously (Hu *et al.* 2017). Quantitative RT-PCR data were derived from 3 independent biological replicates and were analyzed using the comparative method (ΔΔCt). Results were graphed, and the relative expression of each strain was compared to N2. The endogenous control used for normalization was *act-1*. Primer sequences used were described in Hu *et al.* 2017.

### Oxidative stress assays and survival

Paraquat (Methyl viologen dichloride hydrate; Sigma (856177 Aldrich)) was dissolved in H_2_O, kept at -20° and used as required. In experiments involving ROS and gene expression quantification, L4 stage worms were placed in 100 mM paraquat for 1 hr, and then washed with M9 buffer three times. For survival assays, L4 stage worms were transferred to plates containing 2 mM paraquat and scored for survival every day. Worms were transferred to fresh plates every five days. All experiments were performed in three independent replicates.

### ROS quantification assay

The production of ROS was quantified using, 2,7-dichlorodihydrofluorescein-diacetate (DCFDA; Sigma D6883) (Yang *et al.* 2013). The levels of ROS were measured before and after paraquat exposure using a hybrid multimode microplate reader. All strains were synchronized and then incubated with 0 mM or 100 mM paraquat for 1 hr at L4 stage. After incubation, worms were washed with M9 buffer three times, 200 worms/well from each strain (in triplicate) were transferred into each well of a black 96-well plate and mixed with 100 μL of 50 μM DCFDA (diluted in 1X PBS). The fluorescence was measured kinetically every two minutes for 200 min using a BioTek Synergy H4 microplate reader at excitation 485 nm and emission 520 nm, at 25°. To quantify the levels of ROS using confocal microscopy, the worms were incubated for 1 hr in the dark in 1 mL of M9 buffer containing 25 μM DCFDA. After incubation, worms were washed three times with M9 buffer, and images were obtained and then quantified using ImageJ software (National Institutes of Health).

### Statistics

Statistical analysis was performed using Prism 8 software (GraphPad). Statistical significance was determined using an unpaired student’s *t*-test when two means were compared and corrected for multiple comparisons using the Holm-Sidak method. For Figure 5A, the slopes of the curves were fitted using nonlinear regression and compared using one-way Anova as indicated. P values of <0.05 were taken to indicate statistical significance. Error bars represent +/− standard error of the mean.

### Data availability

Strains and plasmids are available upon request. The authors affirm that all data necessary for confirming the conclusions of the article are present within the article, figures, and tables. Supplemental material available at figshare: https://doi.org/10.25387/g3.12624767.

## Results

### The transcription factor SEM-4 is required for phase II detoxification gene expression in brap-2(ok1492) animals and upon oxidative stress

**Figure 1:**
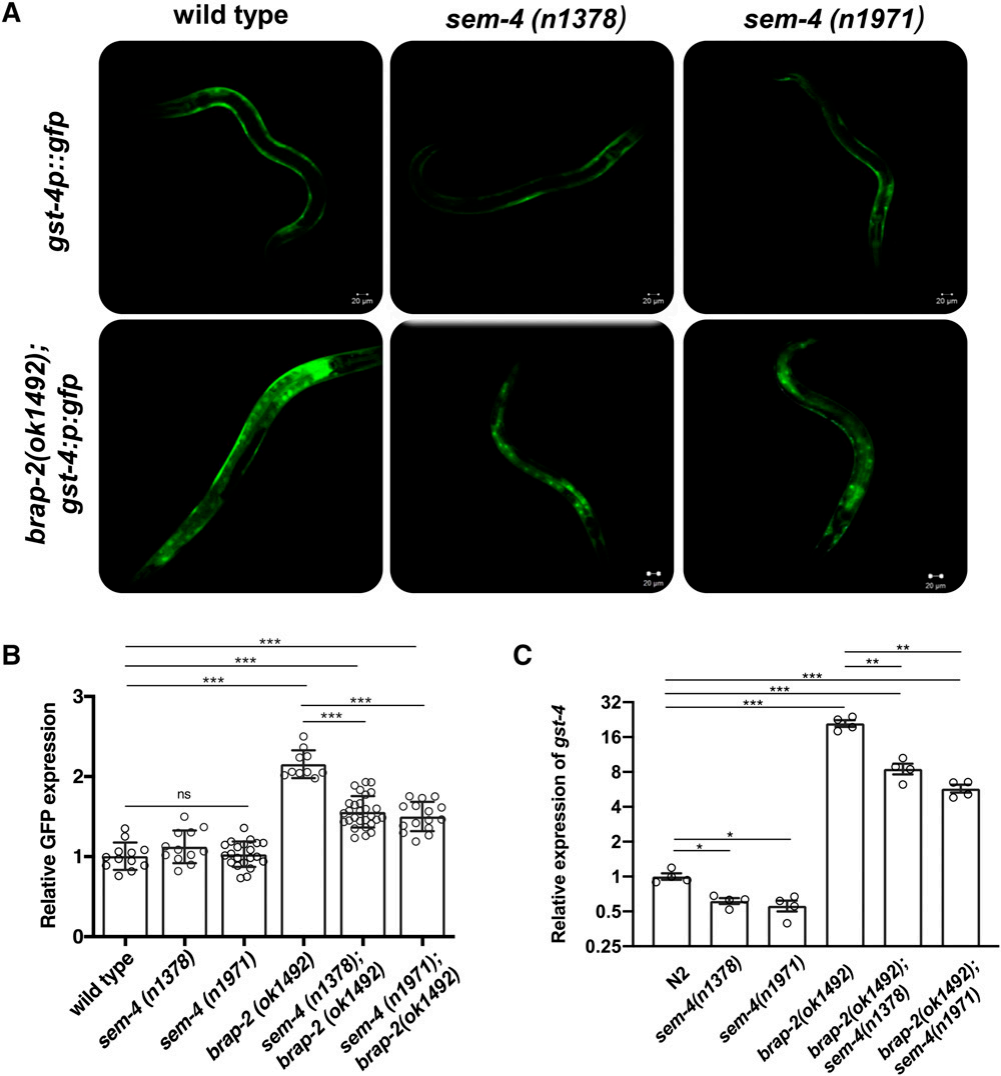
*gst-4* expression is reduced in *sem-4** mutant* worms. (A) GFP expression in *brap-2**(**ok1492**)*; *gst-4p*::*gfp*, *sem-4**(**n1378**)*; *brap-2**(**ok1492**)*; *gst-4p*::*gfp* and *sem-4**(**n1971**)*; *brap-2**(**ok1492**)*; *gst-4p*::*gfp* mutant worms. (B). Images were analyzed with ImageJ software and resulted in significant difference in GFP expression. GFP intensity is lower in both *sem-4* mutant worms (n values between 12 and 28). (C) The *gst-4* mRNA was quantified by quantitative RT-PCR in *sem-4*; *brap-2* mutant worms show significant decrease in comparison to *brap-2* mutant worms. *P* < 0.001***, *P* < 0.01**.

The goal of our lab is to further understand the molecular network of the phase II oxidative stress response. Previously, an RNAi screen identified 18 candidate transcription factors that were required for the enhanced expression of the phase II detoxification gene *gst-4* in the presence of the *brap-2**(**ok1492**)* mutation (Hu *et al.* 2017). SEM-4 was identified in the screen and we set out to validate its role in activating *gst-4* expression. We obtained two strains that carry different mutant alleles of *sem-4*, *sem-4**(**n1378**)* (which contains a C-terminal mutation) and *sem-4**(**n1971**)* (which contains an N-terminal mutation). We generated *sem-4**(**n1378**)*; *brap-2**(**ok1492**)* and *sem-4**(**n1971**)*; *brap-2**(**ok1492**)* mutant worms containing the *gst-4p*::*gfp* transgene and found that the strains containing the *sem-4* mutations showed a weaker GFP expression compared to the *brap-2* single mutant (Figure 1A and 1B).

**Figure 2:**
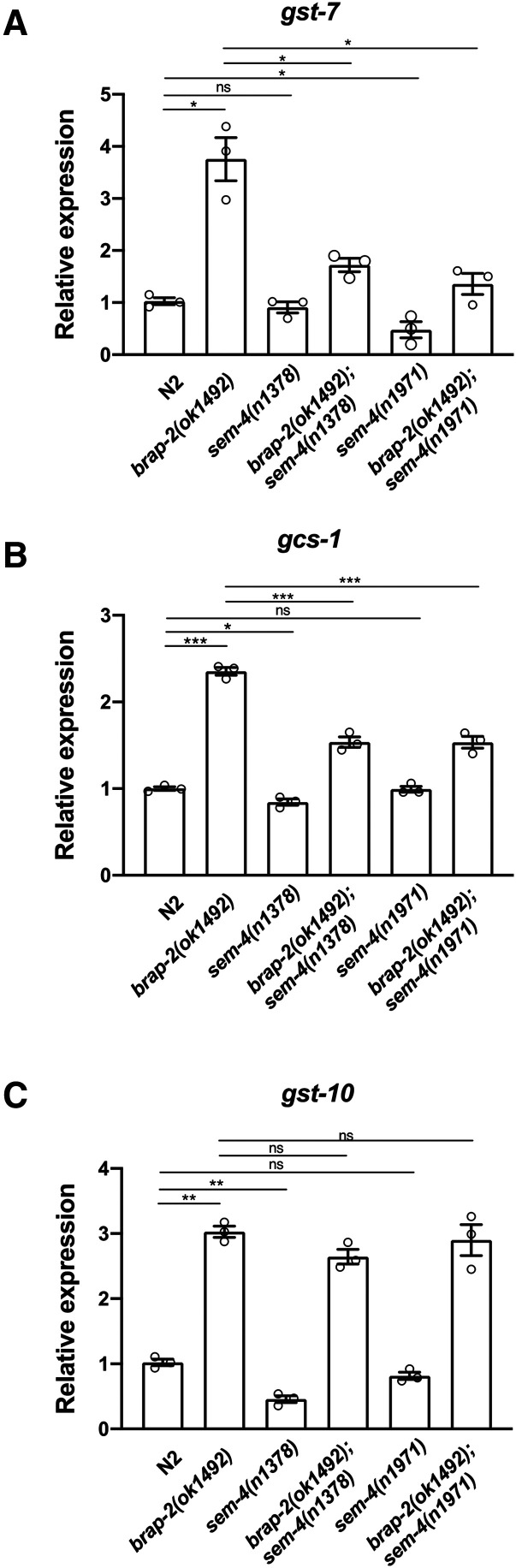
SEM-4 is required to promote phase II detoxification gene expression in *brap-2**(**ok1492**)* worms. The mRNA levels of (A) *gst-7*, (B) *gcs-1* and (C) *gst-10* were quantified using quantitative RT-PCR. The *sem-4**(**n1378**)*; *brap-2**(**ok1492**)* double mutant showed a reduction of mRNA expression for each gene tested. *P* < 0.001***.

We also measured *gst-4* mRNA by quantitative RT-PCR and found that the baseline level of *gst-4* expression was reduced in *sem-4**(**n1378**)* and *sem-4**(**n1971**)* single mutants when compared to wild type. Interestingly, both of the *sem-4*; *brap-2* double mutant strains showed a significant reduction compared to the *brap-2* single mutant (Figure 1C). We quantified the fold induction of *gst-4* by *brap-2**(**ok1492**)* in the presence of the *sem-4**(**n1378**)* or *sem-4**(1971)* alleles and found that while *brap-2* alone induces *gst-4* expression by 20x, in the presence of the *sem-4**(**n1378**)* and *sem-4**(**n1971**)* mutations, *brap-2* enhanced *gst-4* to a lesser extent (13x and 10x respectively, Fig S1). Other phase II detoxification genes (*gst-7*, *gst-10* and *gcs-1*) were assayed and we found that SEM-4 was required for the enhanced levels of expression in *brap-2**(**ok1492**)* for *gst-7* and *gcs-1*, while the *sem-4* mutants did not significantly alter the expression of *gst-10* (Figure 2), possibly due to the existence of other regulating factors specific for *gst-10*.

**Figure 3:**
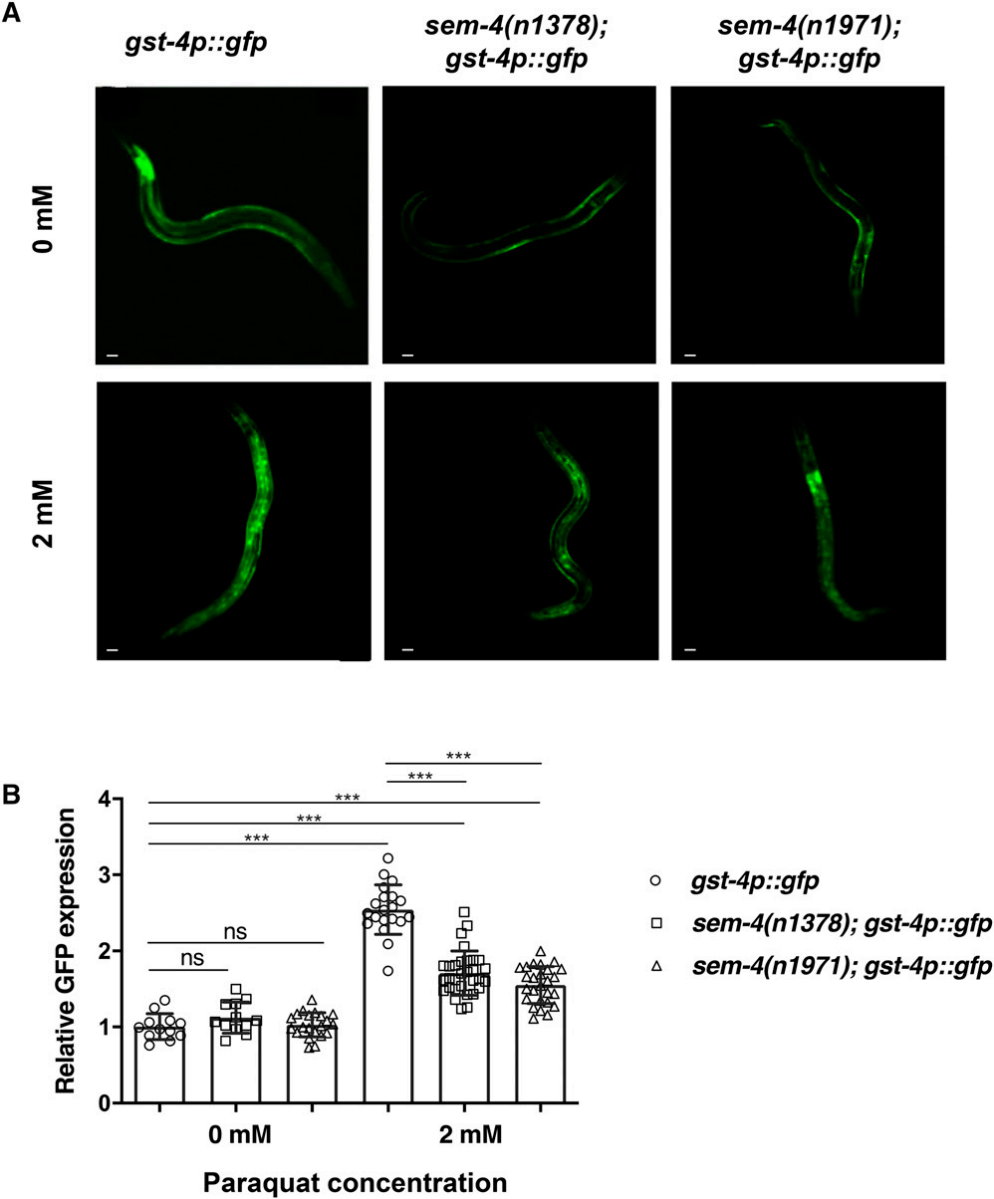
SEM-4 is required to promote *gst-4p*::*gfp* expression during oxidative stress. (A) Fluorescent images of *gst-4p*::*gfp*, *sem-4**(**n1378**)*; *gst-4p*::*gfp* and *sem-4**(**n1971**)*; *gst-4p*::*gfp* worms grown in the absence and presence of 2 mM paraquat. (B) Images were analyzed with ImageJ software and GFP intensity is lower in *sem-4* mutant worms grown on paraquat compared to wild type worms (n values between 12 and 31). *P* < 0.001***, *P* < 0.01**, *P* < 0.05*.

We also assessed the contribution SEM-4 plays during oxidative stress. To do this, we expressed the *gst-4p*::*gfp* transgene in *sem-4**(**n1378**)* and *sem-4**(**n1971**)* single mutants, exposed the worms to paraquat (which induces superoxide formation) and compared their fluorescent levels to wild type worms. We found that in the absence of paraquat, there is no significant difference in *gst-4* promoter activity between wild type and *sem-4* mutants, when the expression of the *gst-4p*::*gfp* transgene was used as a readout of promoter activity. However, upon paraquat exposure, wild type worms demonstrated an increase in GFP levels that were significantly reduced in the presence of either the *sem-4**(**n1378**)* or *sem-4**(**n1971**)* alleles (Figure 3). Together, these results indicate that while functional SEM-4 may be necessary to maintain baseline levels of some phase II detoxification genes, it is required for the induction of a number of these enzymes in the absence of BRAP-2 and upon exposure to oxidative stress.

### SEM-4 regulates expression of skn-1a/c mRNA

To assess if SEM-4 directly interacts with phase II detoxification genes, we surveyed the modENCODE genome-wide ChIP data (Gerstein *et al.* 2010). The five phase II detoxification genes that we surveyed (*gst-4*, *gcs-1*, *gsto-2*, *dhs-8* and *sdz-8*), under basal conditions, showed low levels of interaction of SEM-4 to the promoter regions of these genes (ChIP-seq signal density scores between 10 and 30; Fig S2). Alternatively, we found a relatively higher level of interaction of SEM-4 to *skn-1* (two peaks with a density score of ∼100). Although direct quantitative comparisons between loci may not be valid due to different chromatin accessibility, it does suggest that SEM-4 may influence phase II detoxification gene expression indirectly through regulating *skn-1* gene expression rather than directly interacting with phase II detoxification gene promoters.

**Figure 4:**
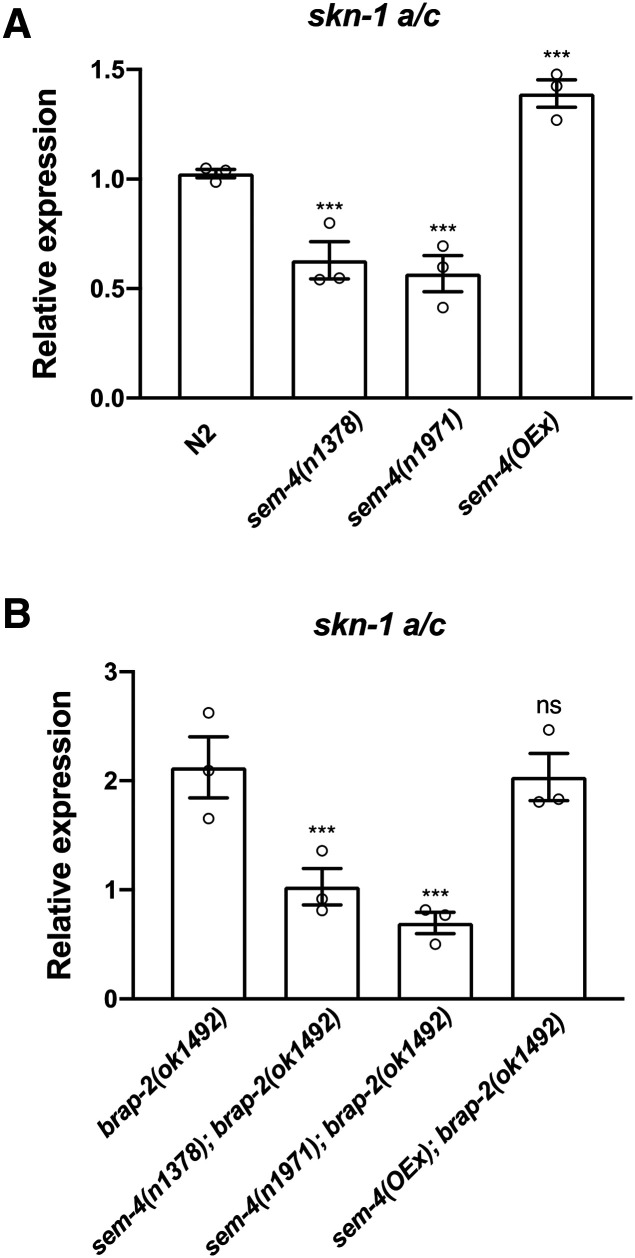
SEM-4 regulates the expression of *skn-1a/c. (*A) mRNA levels of *skn-1a/c* are affected by SEM-4. Overexpression of *sem-4* lead to increase of *skn-1a/c* mRNA by 30% (*P* < 0.001, n = 3 trials), while *sem-4**(**n1378**)* mutant showed a decrease of 40% (*P* < 0.001, n = 5) and *sem-4**(**n1971**)* by 50% (*P* < 0.001, n = 3). (B) SEM-4 regulated expression of *skn-1a/c* in *brap-2* strain. Significantly lower level of *skn-1a/c* mRNA is reported in *sem-4**(**n1971**)*; *brap-2* (65% decrease, *P* < 0.001) and *sem-4**(**n1378**)*; *brap-2* (54% decrease, *P* < 0.001*)* double mutants compared to *brap-2* worms. mRNA levels were quantified by quantitative RT-PCR with *act-1* as a reference gene. *P* < 0.001***.

*C. elegans **skn-1* encodes at least three splice variants (SKN-1A, SKN-1B and SKN-1C), each with its own distinct expression pattern and function. SKN-1A is an ER associated isoform involved in promoting transcriptional response to proteasomal dysfunction while SKN-1B and SKN-1C have roles in caloric restriction and stress resistance, respectively (An and Blackwell 2003; Bishop and Guarente 2007; Glover-Cutter *et al.* 2013). Since SKN-1C is the isoform responsible for regulating phase II detoxification genes in *C. elegans*, we sought to determine if SEM-4 played a role in regulating *skn-1c* expression levels by measuring *skn-1a/c* mRNA levels by quantitative RT-PCR. As done previously (Hu *et al.* 2017), we measured *skn-1a/c* mRNA levels since the complete *skn-1c* exonic sequence is also present in *skn-1a*. We found that under basal conditions, the expression of *skn-1a/c* decreased by 40–50% in *sem-4* mutant worms, while a transgenic strain for expression of SEM-4 (OP57 strain referred to as *sem-4**(OEx)*) showed a 30% increase in *skn-1a/c* mRNA levels (Figure 4A). Since we previously showed that *brap-2**(**ok1492**)* mutants have higher levels of *skn-1* mRNA than wild type animals (Hu *et al.* 2017), we asked if *sem-4* is required for this increased *skn-1a/c* expression. We measured the mRNA levels of *skn-1a/c* in the two *sem-4*; *brap-2* strains and found a decrease in the amount of *skn-1* mRNA compared to the *brap-2* single mutant, restoring it to wild type levels (Figure 4B). This indicates that SEM-4 probably plays a role inducing *skn-1a/c* expression and that the decrease in expression of phase II detoxification genes in *sem-4*; *brap-2* double mutants may be due to the decrease in *skn-1* mRNA levels.

### sem-4 is required to reduce ROS levels in vivo and promote survival upon oxidative stress

**Figure 5:**
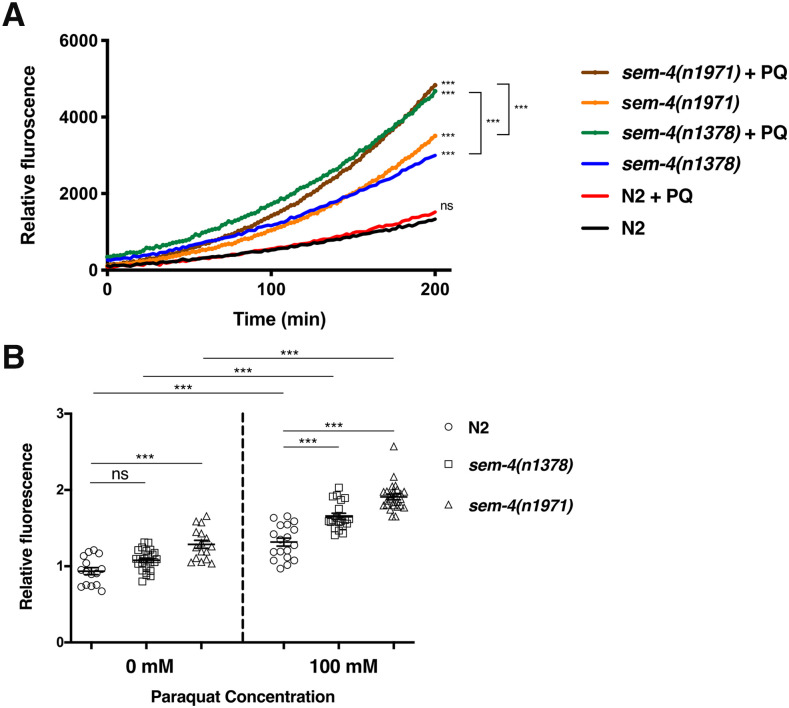
SEM-4 is required to reduce ROS production *in vivo*. Wild type and *sem-4* mutant strains were treated with 0 mM or 100 mM paraquat (+PQ) for 1 h and then mixed with 50 μM DCFDA dye in 96-well plates and the fluorescent levels were read for 200 min. ROS production was recorded as relative fluorescence units (RFU). Both *sem-4** (**n1378**)* and *sem-4**(**n1971**)* showed an increase in ROS production after paraquat treatment over 200 min compared to wild type (N2). (B) Mean results of fluorescence values in presence and absence of 100 mM paraquat (n values between 15 and 25). *P* < 0.001***, *P* < 0.01**.

We also used a functional assay to determine the ability of SEM-4 to increase phase II detoxification enzyme levels and reduce ROS *in vivo*. To do this, we quantified ROS levels using 2,7-dichlorodihydrofluoresceindiacetate (DCFDA) dye in three strains: N2, *sem-4**(**n1378**)*, and *sem-4**(**n1971**)* upon exposure to 0 or 100 mM paraquat. As expected, we observed a significant increase ROS production in both *sem-4* strains when treated with paraquat in comparison to N2 (Figure 5A). The ROS production levels in the three strains were further evaluated using confocal microscopy. Fluorescence intensities were quantified and showed the highest ROS production being detected in *sem-4**(**n1971**)* and followed by *sem-4**(**n1378**)* mutants (Figure 5B). The lowest ROS levels were recorded in N2 worms. Therefore, the loss of *sem-4* elevates ROS production following oxidative stress induction, demonstrating its importance in regulating the detoxification of ROS.

**Figure 6:**
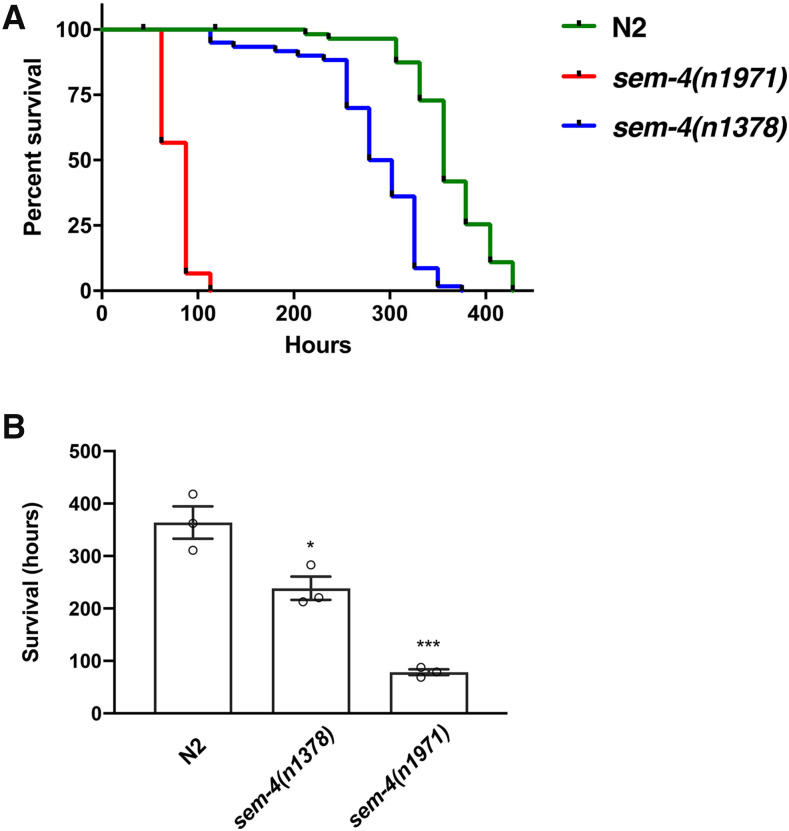
Survival following oxidative stress is dependent on SEM-4. (A) Survival of three strains were tested on 2 mM paraquat plates showed necessity of SEM-4 for *C. elegans*. The shortest lifespan is observed in *sem-4**(**n1971**)*, mean lifespan is 79 hr. The longest survival is observed in wild type worms, mean lifespan is 363 hr, and *sem-4**(**n1378**)* worms survived for 283 hr. (B) Mean results for three trials. Survival of *sem-4**(**n1971**)* is decreased by 78% (*P* < 0.001) in comparison to N2 and survival of *sem-4**(**n1378**)* is decreased by 34% (*P* = 0.0303). Data were analyzed using OASIS software. *P* < 0.001***, *P* < 0.05*.

To test the importance of SEM-4 on the survival of worms following oxidative stress, we cultured *sem-4* mutant nematodes on plates containing paraquat. For this experiment, *sem-4**(**n1971**)*, *sem-4**(**n1378**)* and N2 worms were grown to L4 stage and transferred to 2 mM paraquat + 0.05 mg/mL FUDR plates and observed daily (Figure 6). It was found that *sem-4**(**n1971**)* worms median survival rate at 79 hr, while the median for *sem-4**(**n1378**)* and N2 were at 239 hr and 363 hr respectively (Figure 6B). These results demonstrate that the loss of functional SEM-4 is detrimental for worm survival when exposed to paraquat and that the inability of *sem-4* worms to survive in oxidative stress conditions corresponds to the detection of very high levels of ROS *in vivo*, although we do have to acknowledge the possibility that the FUDR present in the assay may have varying effects on the oxidative stress response of the different strains (Park *et al.* 2017).

## Discussion

We have previously shown that BRAP-2 regulates PMK-1/p38 in *C. elegans* (Hu *et al.* 2017). Upon oxidative stress in a *brap-2* mutant strain, the activated PMK-1 phosphorylates SKN-1C to promote its re-localization from the cytosol to the nucleus and promotes phase II detoxification genes expression. Our work here indicates that the transcription factor SEM-4 is also required in response to oxidative stress, probably through the regulation of *skn-1c* gene expression, although we cannot rule out that SEM-4 may directly bind to phase II detoxification gene promoters. For this study, we used two alleles of *sem-4*, *n1378* and *n1971*, with the *n1971* allele considered to be the stronger allele (Basson and Horvitz 1996). We found that both alleles generate similar results when we assayed for phase II gene expression; however, the *n1971* animals were seen to be more sensitive to stress in the survival assay. While both alleles may affect the SKN-1 detoxification pathway, our results indicate that the *n1378* allele may produce a form of SEM-4 capable of providing some protective function *in vivo*, perhaps by activating other parallel pathways. Although this study investigates SEM-4 role in phase II oxidative stress response, further work is required to determine if SEM-4 also involved in other stress response pathways.

Like SKN-1, SEM-4 was initially discovered based on its essential role in development (Basson and Horvitz 1996), and our results show that SEM-4 also has a role in maintaining cellular homeostasis in terminally differentiated cells. RNAseq experiments have indicated that both *skn-1* and *sem-4* mRNA are present in all lineages (mesoderm, endoderm, ectoderm and germline) during development (Hashimshony *et al.* 2015), but less data for SEM-4 levels in different tissues is available for larval or adult stages. While SKN-1A/C has been shown to be expressed in adult intestinal cells, SKN-1 can induce detoxification gene expression in the hypodermis (Paek *et al.* 2012). Although we have not identified the adult tissue(s) within which SEM-4 functions, it is conceivable that SEM-4 and SKN-1 are functional in tissues when expressed at low levels.

This study also suggests that the mammalian orthologs of SEM-4 may be involved in the oxidative stress response. There are four known mammalian genes: SALL1/2/3/4 that share homology with SEM-4 and, to our knowledge, none of these gene products are associated with oxidative stress. There are known diseases associated with mutations in different SALL genes in humans. For instance, a mutation in SALL1 is linked to the autosomal disease, called Townes-Brocks syndrome (Netzer *et al.* 2006), while mutant forms of SALL2 are present in human ovarian carcinoma (Toker *et al.* 2003) and a mutation in SALL4 leads to Okihiro syndrome (Duane-radial ray syndrome) (Kohlhase *et al.* 2005). The function of SALL3 was discovered as an inhibitor of DNA methylation (Shikauchi *et al.* 2009). Therefore, we anticipate that this study will provide a framework for further investigation of SALL1/2/3/4 in the mammalian oxidative stress response that may play a pathological role in a number of tissues and human diseases.

While the detrimental effects of ROS on the function of biological macromolecules is believed to lead to aging and cell death, ROS also acts as a fundamental signaling molecule in numerous processes, with hydrogen peroxide and singlet molecular oxygen having significant second messenger roles (Shibata *et al.* 2003; Putker *et al.* 2013; Holmström and Finkel 2014; Sies *et al.* 2017). The interplay between excessive oxidative conditions and maintenance of appropriate physiological levels of ROS requires the regulation of levels of detoxification enzymes by stress responsive transcription factors (Sies and Jones 2020). Interestingly a number of transcription factors that have previously been demonstrated to play important developmental roles, such as *skn-1*, *elt-3* and *sem-4*, appear to have been repurposed in differentiated cells to function as regulators of ROS signaling. Further studies are required to understand how these (and other) factors co-ordinate together to properly regulate ROS levels.
